# The experience of admission to psychiatric hospital among Chinese adult patients in Hong Kong

**DOI:** 10.1186/1471-244X-8-86

**Published:** 2008-10-17

**Authors:** Jackie Chi-Kin Fu, Paulina Po-Ling Chow, Linda Chiu-Wa Lam

**Affiliations:** 1 General Adult Team, Department of Psychiatry, Castle Peak Hospital, Tuen Mun, Hong Kong, PR China; 2 Psychogeriatric Team, Department of Psychiatry, Castle Peak Hospital, Tuen Mun, Hong Kong, PR China; 3 Department of Psychiatry, the Chinese University of Hong Kong, Tai Po Hospital, Tai Po, Hong Kong, PR China

## Abstract

**Background:**

The paper reports on a study to evaluate the psychometric properties and cultural appropriateness of the Chinese translation of the Admission Experience Survey (AES).

**Methods:**

The AES was translated into Chinese and back-translated. Content validity was established by focus groups and expert panel review. The Chinese version of the Admission Experience Survey (C-AES) was administered to 135 consecutively recruited adult psychiatric patients in the Castle Peak Hospital (Hong Kong SAR, China) within 48 hours of admission. Construct validity was assessed by comparing the scores from patients admitted voluntarily versus patients committed involuntarily, and those received physical or chemical restraint versus those who did not. The relationship between admission experience and psychopathology was examined by correlating C-AES scores with the Brief Psychiatric Rating Scale (BPRS) scores.

**Results:**

Spearman's item-to-total correlations of the C-AES ranged from 0.50 to 0.74. Three factors from the C-AES were extracted using factor analysis. Item 12 was omitted because of poor internal consistency and factor loading. The factor structure of the Process Exclusion Scale (C-PES) corresponded to the English version, while some discrepancies were noted in the Perceived Coercion Scale (C-PCS) and the Negative Pressure Scale (C-NPS). All subscales had good internal consistencies. Scores were significantly higher for patients either committed involuntarily or subjected to chemical or physical restrain, independent on severity of psychotic symptoms.

**Conclusion:**

The Chinese AES is a psychometrically sound instrument assessing the three different aspects of the experience of admission, namely "negative pressure, "process exclusion" and "perceived coercion". The potential of C-AES in exploring subjective experience of psychiatric admission and effects on treatment adherence should be further explored.

## Background

Compulsory treatment has been one of the most debated issues in psychiatry [1]. In Hong Kong, compulsory psychiatric treatment is justified for any person who suffers from a psychiatric disorder and poses danger to oneself or others. However, removal of personal liberty under the doctrine of *parens patriae *raises questions over ethical and legal justifications. In addition, compulsory treatment may lead to the perception of coercion [2-4], which may affect patient's attitudes and their adherence to treatment [5,6]. Before attempting to study the effects of compulsory treatment itself, it is important to gain more insight into the beliefs and attitudes of patients regarding the experience of the admission process.

Research related to coercion in the context of psychiatry flourished in the 1990s with the advent of instruments for the quantification of perceived coercion during psychiatric admission. The Admission Experience Survey (AES) was developed by the MacArthur Research Network on Mental Health and the Law [7] to assess psychiatric patients' global perceptions of their hospitalization. Originating from the structured Admission Experience Interview [7], it was simplified to a fifteen-item true/false statement format. The AES has been widely used and applied across different cultural boundaries, [4,8] and adapted to evaluate out-patient commitment programmes [9,10]. Three subscales have been derived from this instrument, including the five-item Perceived Coercion Scale, which has been found to be a psychometrically sound and internally reliable measure of patient's perception of coercion [2,7] with satisfactory retest stability [11]. It has also been incorporated into cross-nation multi-centre studies [4,12-14]. The second scale was the six-item Perceived Negative Pressure Scale. The term "Negative Pressure" refers to the pressure that indicates a worse consequence if one were to resist, such as the use of threats or physical forces, whereas "Positive Pressure" refers to more benign actions such as persuasion and inducement [5,15,16]. The four-item Process Exclusion Scale measured patients' evaluation of the fairness of admission process, which is related to their perceptions of others' motives and whether their views being considered [5]. Both process exclusion and negative pressure have been found to predict perceived coercion [15]. Despite healthcare professionals appeared to be the main source of pressure [17], it has been pointed out that if patients perceived they were treated in good faith with impartiality, they were more likely to view the hospital admission as fair and less likely to perceive coercion [1,18].

Coercion is legitimately context-dependent [19] and justification of medical practices and civil commitments are subject to differences in cultures and beliefs. Therefore, it deemed necessary to explore the influence of different cultures on coercion in psychiatric admission. Research in this aspect in Chinese patients has been impeded by the lack of a standardized, culturally-adapted instrument. It is hoped that validating the Chinese version of the Admission Experience Survey (C-AES) would allow meaningful comparisons of results across different cultures, and enable future study on the amenable factors of psychiatric service that may affect admission experience.

## Methods

This project was approved by the Clinical & Research Ethics Committee of New Territories West Cluster, Hospital Authority (Hong Kong SAR, China) and complies with the 1996 version of the Declaration of Helsinki. Standard psychometric procedures were employed, aiming at producing a cross-culturally compatible version of the AES. The project consisted of a qualitative stage followed by a validation stage, with the results of the first stage informing the next. Both qualitative and quantitative methods were incorporated to allow a richer view in the current research of perceived coercion [18].

### Qualitative stage

The AES was translated into written Chinese and back-translated by independent postgraduate linguists fluent in English and Chinese. The C-AES was then evaluated in focus groups followed by expert panel review. Patients with different diagnoses including psychotic disorders, mood disorders, anxiety disorders, substance misuse disorders and personality disorders, and particularly those with a history of hospitalization, were selectively recruited into the focus groups to improve the representativeness of the sample. All conversations were recorded electronically. Non-verbal interactions were recorded manually. Typed transcripts and field notes were reviewed and generated into themes, making possible the comparison of the differences and similarities of all cases based on the data obtained. The results provided the basis to measure the face and construct validity of the C-AES.

After introduction and obtaining consent, participants were invited into focus groups. Introductory questions were put out to assist the participants to acclimatize to the atmosphere. Topics discussed included the definition of coercion, the actual context in which coercion took place, description of actual scenarios in their own accounts, and their subjective feelings. Researches on coercion in psychiatric admission have reported that involuntary admission conferred more coercive feelings [2-4,20]. Whether this is a universal perception in the Chinese community was explored through the views of participants. Guided discussions served to ascertain from patients' perspective, the linkage of objectively measurable coercive measures and subjectively perceived coercion. Participants were then issued the C-AES for their comments on the coverage of the C-AES, the readability, clarity of the instructions and items, and how the statements related to their concepts and experience of coercion. Fine adjustment to the questionnaire was made accordingly.

The C-AES was then evaluated by an expert panel comprising twelve medical staff having more than ten years of experience in public psychiatric service, including consultant psychiatrists, clinical psychologists, occupational therapists, psychiatric nurses and social workers. An expert panel questionnaire was completed by each panel member, which included definitions of each of the items and five-point Likert scales, rating the respective relevance to the definition. An open-ended box was provided for respondents to give their reasons of ratings and suggestions.

### Validation stage

The validation stage was a cross-sectional study conducted in Castle Peak Hospital (CPH), a tertiary unit in Hong Kong, providing psychiatric in-patient services for the Tuen Mun and Yuen Long areas, serving a population over one million [21]. All patients aged between 18 and 65 years admitted for the treatment of a new episode of psychiatric illness were consecutively recruited. Any patient admitted more than once within the recruitment period would only have the first admission counted. Patients with organic diagnoses including mental retardation, dementia or organic brain diseases were excluded due to the lack of mental capability to give consent to the study and to comprehend the questionnaire. Sample size was determined to be 135, based on the recommended item-to-response ratio range of 1:4 to 1:10 determined from the literature [22]. Sampling adequacy was further examined by Kaiser-Meyer-Olkin measure.

A pilot batch of C-AES questionnaires were administered to 15 newly-admitted patients, following the recommended pre-test size range [23]. Their views and responses were used to refine the interview and workflow. After the pilot test, all patients admitting from 9^th ^February to 15^th ^April 2008 fulfilling the recruitment requirements were recruited. Written informed consents were obtained from participating patients. Patients refused to join the study would have their verbal consent sought to record their demographic data. All interviews were conducted within the first 48 hours of admission to allow a reliable measurement of perception of coercion [11]. Case notes were reviewed and collateral informants were either interviewed or contacted by telephone. To minimize recall bias, patients' accounts were triangulated with case notes and collateral informant interviews. Particular attention was paid to documentation of prescriptions or restraint instructions in medical notes. Any subsequent inconsistencies were resolved according to a set of hierarchically-arranged rules adapted from the study by Lidz *et al *in which the "most plausible factual account" was defined [15]:

1. Always believe an individual's own account of his or her motives rather than someone else's account. Questioning of this account must be based on inconsistent objective data, not on someone else's account of motives for agreed-upon acts.

2. Believe an eyewitness account before a second-hand report.

3. Accept the fuller account of an incident rather than the sparser one.

4. If the preceding rules do not yield a choice of account, believe multiple sources before a single source.

The C-AES was issued to each patient after obtaining consent. Questionnaires were coded anonymously to alleviate subjects' concern of their results being individually identifiable. Anonymously-coded C-AES was issued to each participant to alleviate their concern of results being individually identifiable. Finished questionnaires were sealed in envelopes and blinded to the interviewer to minimize observer bias. Subjects were then given an extensive diagnostic interview. All diagnoses were made by consensus with the attending psychiatrist and consultant psychiatrists according to International Classification of Diseases version 10 classification of mental and behavioural disorders [24]. Psychiatric symptoms were rated using the Brief Psychiatric Rating Scale (BPRS) [25].

Using the known group method, the theoretical context behind the construct concluded from focus groups was used to predict how different patient groups were expected to behave. Based on focus group results, it was postulated that patients involuntarily committed and those who had been subjected to "objective coercive measures" (OCM) (see Qualitative stage focus group results) would have higher C-AES scores. Scores from respective groups were compared statistically. Whether paranoid symptoms had conferred the feeling of coercion to psychotic patients was unknown. Therefore an established instrument for psychotic symptom screening, the BPRS, was used to examine any correlation between the severity of psychotic symptoms and C-AES scores. BPRS has been shown to be a reliable tool for the assessment of psychotic symptoms in various clinical diagnoses [25,26].

### Statistics

Data were analyzed using the Statistical Package for the Social Sciences for Windows Version 16. Data normality was checked using Kolmogorov-Smirnov test. Since the C-AES was not normally distributed, non-parametric statistical methods were used. Item-to-total correlations were calculated by using Spearman rank correlation coefficients. Individual items of C-AES were tested for association with whether patients were subjected to "objective coercive measures" by Spearman rank correlation. C-AES scores between voluntarily admitted versus compulsorily admitted subjects, and those subjected to objective coercive measures versus those not, were compared by Mann-Whitney *U *test.

Exploratory factor analysis was performed to examine the constructs. Principal component analysis was performed to extract factors with eigenvalues greater than 1.0. Orthogonal rotation by Varimax method was used for identification of uncorrelated factors [27].

The relationship between admission experience and psychotic symptom severity was examined through Spearman's correlations between BPRS, C-AES and the respective subscales.

Item 12 of the C-AES – "No one tried to force me to come into the hospital" was written in the reverse to item 2 – "People tried to force me to come into the hospital". Subsequent statistics showed that item 12 had low item-to-total correlation (see Validation stage results). Relationship between a discordant response to items 2 and 12 and clinical variables were analyzed using binary logistic regression, including sex, age, number of previous voluntary or compulsory admission, length of disease, diagnosis, BPRS scores, and C-AES subscales. It was hypothesized that those factors related to cognitive function would be significant predictors.

## Results

### Qualitative stage

Thirty-two participants provided opinions in focus groups, of which ten were in-patients, fourteen were out-patients and eight were relatives of patients. The main theme of the focus groups was first defined by introducing the term "coercion" within the context of "psychiatric admission". In Chinese translation "coercion" literally means "to make somebody do something by using force or threat". All participants agreed that the concept of coercion existed in psychiatric admission in Hong Kong. Participants were further invited to elaborate on their own concept of "coercion". They generally perceived that involuntary commitment conferred more "coercive feeling" than voluntary admission. Such "coercive feeling" originated from external pressure by police, medical staff, family members or any significant others, in the form of attitude, use of language, gesture or physical force. Participants had different descriptions about coercion, which could be generalized into objective and subjective dimensions. From a subjective perspective, participants in general viewed "coercion" as the feeling of deprivation of autonomy, the fear from threats during the admission process and unknown consequences which some misconceived to be legal in nature. From an objective point of view, participants frequently referred to observable behaviour such as seclusion, sedative injection and physical restraint. All participants agreed on the reiteration that coercion could be categorized into subjective and objective dimensions. They were further asked for their views on any link between the two dimensions. Some suggested a causal relationship such that the exercise of certain coercive measures that would "unavoidably" lead to the perception of coercion by others. Such measures including verbal threats, assembling security guards to standby, chemical sedative measures and physical restraint, which were generally agreed by most participants. As the latter two could readily and objectively be accessible through triangulation of case notes with the accounts of patients and collateral informants, such information were collectively defined as "objective coercive measures" (OCM) for the assessment of the construct validity of the C-AES. Triangulation of views from multiple informants has been considered to be essential for coercion studies [1,18]

Considering the C-AES questionnaire, all participants agreed on the scope of coverage on admission experience, appropriateness of use of language and length of the questionnaire, although item 2 and 12 appeared redundant. Participants also suggested alternative wordings on the translation of item 1 and 10, and suggested the translation of the term "threatened with commitment" in item 10 into a literal meaning of "threatened by applying law for compulsory admission" in Chinese.

After five sessions no further information could be generated and data saturation was concluded to have been achieved. All translation and alteration by focus group suggestions were examined together with the original author. Expert panel review endorsed all translations and modifications.

### Validation stage

A pilot test on 15 newly admitted in-patients confirmed the readability of the questionnaires and feasibility of the structure of the interview. Two five-minute breaks within the approximately one-hour interview were considered necessary to maintain patients' attention span.

To identify OCM, information from patient interview was triangulated with case notes and collateral informant interviews. Conflicting accounts were rare and invariably referred to motives rather than the nature of actions actually taken, which were resolved after applying the hierarchically-arranged rules.

**Figure 1:**
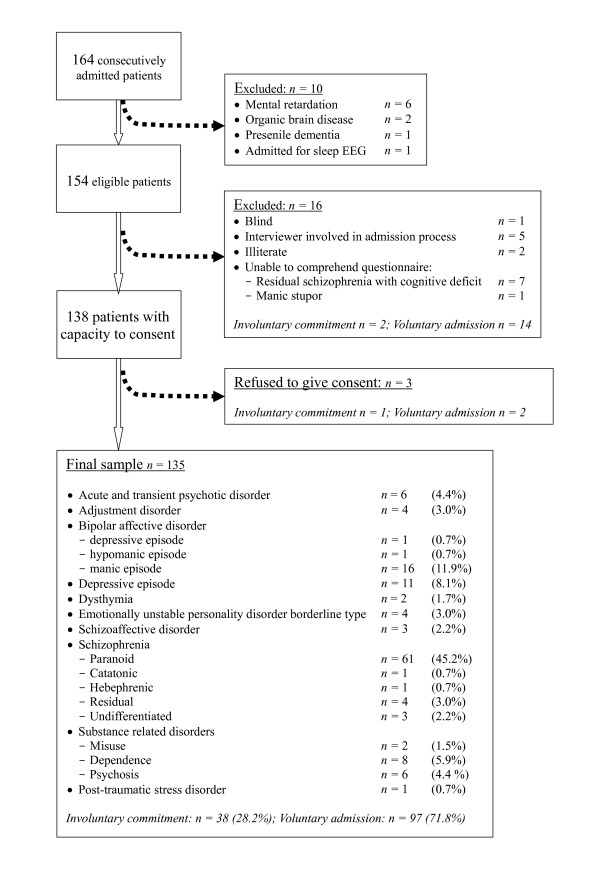
Flowchart of subject recruitment.

A total of 164 consecutive admissions were analyzed. Of these, 154 patients were considered eligible after excluding diagnoses of organic brain diseases, dementia and mental retardation. Another 16 patients (10.4%) were excluded due to difficulty in obtaining an informed consent, illiteracy or visual impairment. Three patients (1.9%) refused to give consent to the study, but agreed to have their demographic data recorded. The final sample comprised 135 patients [Figure 1]. The mean age was 38.19 (standard deviation = 11.81) and female to male ratio was 1:1.18. The median length of mental illness was 10 years (range = 0 – 41). The median number of years of education was 12 (range = 0 – 20). Maximum number of previous admission was 25 (median = 1). In view of the small number of subjects of those refused to consent, statistical comparison of the differences between their characteristics with the study sample was omitted.

**Figure 2:**
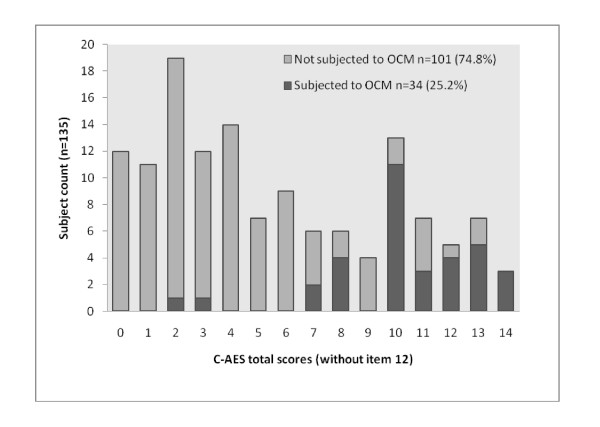
**Distribution of C-AES scores by exposure to OCM**. *C – AES*, Chinese version of Admission Experience Survey; *OCM*, Objective coercive measures.

**Figure 3:**
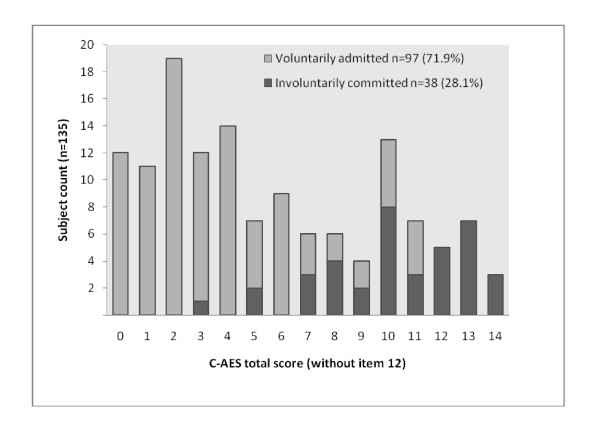
**Distribution of C-AES by legal status**. *C – AES*, Chinese version of Admission Experience Survey.

The C-AES score ranged from 0–15, with a mean score of 6.06 (S. D. = 4.29). The score did not conform to normality with kurtosis -1.07 and skewness 0.41. Homogeneity of the scale verified by item-to-total correlations showed that all items had moderate to good correlations with the exception of item 12. Spearman's *ρ *ranged from 0.31 to 0.73. When item 12 was excluded from calculation, Spearman's *ρ *ranged from 0.50 to 0.74. Distribution of score was bimodal, with kurtosis and skewness of -1.09 and 0.43 respectively [Figures 2, 3]. When sub-group C-AES scores were analysed separately according to participants' legal statuses and being exposed to OCM or not, individual distributions did not conform to normality although the bimodal distribution was not observed.

Exploratory factor analysis was performed to examine the constructs of C-AES. Kaiser-Meyer-Olkin Measure was 0.85, consistent with good sampling adequacy. Barlett's test of sphericity reached statistical significance for factorability. Employing principal component analysis, three factors were identified after Varimax rotation. All items except item 12 clustered into the three factors. Since item 12 showed poor factor loading and item-to-total correlation, it was excluded from subsequent calculations. Items 2, 4, 6, 7, 8, 10 and 11 were grouped under factor 1, covering 27.12% of the total variance. This factor covered the pressure on patients that they perceived as negative in nature, and was designated as the Chinese version of Negative pressure scale (C-NPS). The rotated component loadings varied from 0.69 to 0.77. Factor 2 comprised items 3, 5, 9 and 13, covering 17.95% of the total variance. This factor structure concurred with the "Process Exclusion Scale" (PES) coined by Hiday et al [5], referring to the perceived lack of validation and voice during the admission process. This factor was designated as the Chinese version of Process Exclusion Scale (C-PES). The rotated component loadings varied from 0.70 to 0.77. Factor 3 comprised items 1, 14 and 15, covering 16.77% of the total variance. It measures patients' perceived coercion over coming to the hospital, and was designated as the Chinese version of "Perceived coercion scale" (C-PCS). The rotated component loadings varied from 0.67 to 0.83. The three factors explained 61.83% of total variance. The Cronbach's alpha for Factors 1, 2 and 3 were 0.88, 0.77 and 0.74 respectively, consistent with good internal consistency [Table 1].

**Table 1:** Factor loading of C-AES.

		Varimax-rotated component loadings		
		
Items		Factor 1	Factor 2	Factor 3
	**Factor 1 **(Negative pressure scale, *Cronbach's α = 0.88*)			
6	Someone threatened me to get me to come into the hospital	0.77		
10	I was threatened with commitment	0.75		
2	People tried to force me to come into the hospital.	0.75		
8	Someone physically tried to make me come into the hospital	0.70		
4	I chose to come into the hospital	0.69		
11	They said they would make me come into the hospital	0.69		
7	It was my idea to come into the hospital	0.60		
				
	**Factor 2 **(Process exclusion scale, *Cronbach's α = 0.77*)			
5	I got to say what I wanted about coming into the hospital		0.77	
3	I had enough of a chance to say whether I wanted to come into the hospital		0.76	
9	No one seemed to want to know whether I wanted to come into the hospital		0.72	
13	My opinion about coming into the hospital didn't matter		0.70	
				
	**Factor 3 **(Perceived coercion scale, *Cronbach's α = 0.74*)			
14	I had a lot of control over whether I went into the hospital			0.83
15	I had more influence than anyone else on whether I came into the hospital			0.77
1	I felt free to do what I wanted about coming into the hospital			0.67
				
Eigenvalues (Varimax-rotated)		3.51	2.51	2.35
Percentage of variance (Varimax-rotated)		27.12	17.95	16.76

Item 12 was omitted. Factors with eigenvalues > 1 were retained. Factor loadings < 0.5 were omitted. Cronbach's *α *for each subscale provided for reference.

C-AES and subscales were all found to be significantly higher on subjects involuntarily committed and subjects exposed to OCM [Table 2]. Among all items of the C-AES, item 8 – "Someone physically tried to make me come into the hospital" had the highest correlation with patients who had been subjected to OCM (Spearman's *ρ *= 0.78, *p *< 0.001) [Table 3]. No significant correlation was found between C-AES and BPRS or psychotic sub-score [Table 4].

**Table 2:** Comparison of C-AES and subscales between different patient groups.

		**Legal statuses**		**Exposure to OCM**	
			
		Involuntarily committed *n *= 38	Voluntarily admitted *n *= 97	Exposed *n *= 34	Not exposed *n *= 101
			
**C-AES**	Mean (S.D.)	10.26 (2.73)	3.77 (3.01)	10.26 (2.79)	4.03 (3.29)
	Mean rank	109.29	51.82	108.75	54.28
	Mann-Whitney Effect size (*r*)	0.66**		0.61**	
**NPS**	Mean (S.D.)	5.34 (1.51)	1.21 (1.73)	5.09 (1.75)	1.46 (2.00)
	Mean rank	110.03	51.54	54.85	107.06
	Mann-Whitney Effect size (*r*)	0.69**		0.60**	
**PES**	Mean (S.D.)	2.37 (0.94)	1.18 (1.14)	2.50 (0.83)	1.18 (1.34)
	Mean rank	94.67	57.55	57.55	99.04
	Mann-Whitney Effect size (*r*)	0.44**		0.48**	
**PCS**	Mean (S.D.)	2.55 (1.52)	1.39 (1.38)	2.68 (1.53)	1.40 (1.36)
	Mean rank	88.30	60.05	60.30	90.87
	Mann-Whitney Effect size (*r*)	0.33**		0.35**	

Mean values of score provided for reference.

*C-AES*, Chinese version of Admission Experience Survey; *NPS*, Negative Pressure Scale; *PES*, Process Exclusion scale; *PCS*, Perceived Coercion Scale; *OCM*, Objective coercive measures.

**P < 0.01 (2-tailed)

**Table 3:** Correlations of OCM with C-AES items.

C-AES Item		Spearman's *ρ*
8	Someone physically tried to make me come into the hospital	0.78**
1	I felt free to do what I wanted about coming into the hospital	0.52**
4	I chose to come into the hospital	0.51**
2	People tried to force me to come into the hospital.	0.48**
7	It was my idea to come into the hospital	0.47**
6	Someone threatened me to get me to come into the hospital	0.46**
10	I was threatened with commitment	0.40**
3	I had enough of a chance to say whether I wanted to come into the hospital	0.36**
15	I had more influence than anyone else on whether I came into the hospital	0.36**
5	I got to say what I wanted about coming into the hospital	0.35**
14	I had a lot of control over whether I went into the hospital	0.29**
11	They said they would make me come into the hospital	0.27**
13	My opinion about coming into the hospital didn't matter	0.22
9	No one seemed to want to know whether I wanted to come into the hospital	0.21
12	No one tried to force me to come into the hospital.	0.18

*n *= 135.

*C – AES*, Chinese version of Admission Experience Survey; *OCM*, Objective coercive measures.

**P < 0.01 (2-tailed)

**Table 4:** Correlations of C-AES, BPRS and respective subscales.

	Spearman's *ρ*				
	
	**BPRS**	**BPRS-P**	**BPRS-M**	**BPRS-D**	**BPRS-O**
**C-AES**	-0.02	0.06	0.09	-0.18	0.15
**NPS**	-0.08	0.00	0.062	-0.22	0.09
**PES**	0.11	0.16	0.155	-0.08	0.13
**PCS**	-0.08	0.00	-0.01	-0.12	0.14

*n *= 135

*C-AES*, Chinese version of Admission Experience Survey; *NPS*, Negative Pressure Scale; *PES*, Process Exclusion scale; *PCS*, Perceived Coercion Scale; *BPRS*, Brief Psychiatric Rating Scale; *BPRS-P*, Brief Psychiatric Rating Scale total psychotic subscore; *BPRS-M*, Brief Psychiatric Rating Scale total manic subscore; *BPRS-D*, Brief Psychiatric Rating Scale total depressive subscore; *BPRS-O*, Brief Psychiatric Rating Scale total other subscore.

Responses to item 12 had the lowest item-to-total correlation and loading in factor analysis. Logistic regression showed that a disparity of responses to items 2 and 12 was predicted by fewer years of education (Odds ratio = 1.12, 95% confidence interval 1.07 – 1.17) and more years of illness (Odds ratio = 0.76, 95% confidence interval 0.66 – 0.86).

## Discussion

This is the first study known to the authors to investigate the admission experience of Chinese patients. The AES was translated into Chinese and validated using standardized psychometric methods. All items, except item 12 showed acceptable item-to-total correlations.

The C-AES followed a bimodal distribution, a phenomenon invariably reported in other studies [5,7,12,20]. One postulation is that while the survey is devoid of any objective elements, it represents a pure measurement of subjective dimensions of patients' experience. This makes this instrument by large a rating of patients' emotional component and hence a dichotomized pattern of response was observed.

Responses to item 12 had the lowest item-to-total correlation and loading in factor analysis. One of the themes generated from the focus groups was about the redundancy of this item with item 2. This item was written as the exact reverse of item 2, requiring it to be answered in a negative fashion. It was possible that some of the subjects might have over-looked the item, or misinterpreted the question as if they were asked whether anyone tried to force them to come into the hospital. Logistic regression revealed that less education and longer period of mental illness had a detrimental effect on the interpretation and appraisal of item 12, although how cognitive functions would affect admission experience remains unclear. Future users of this instrument could consider omitting item 12.

Exploratory factor analysis was performed to examine the construct of the scale. While a factor structure approximating the original scales permits international comparison of results and better applicability, there existed some differences between the constructs of C-AES and the original English version. Item 4 "I chose to come into the hospital" and item 7 "It was my idea to come into the hospital" were loaded on the "Negative Pressure Scale" instead of "Perceived Coercion Scale". Giving a response to these two items required participants to commit whether they were the sole decision-makers for hospital admission. Traditionally, the Chinese mentality is influenced by Confucianism, which emphasizes the "appropriateness" of interpersonal relationships [28]. Physicians are expected to assume parental roles [29] and they might have a tendency to become assertive when offering treatment options. Alternatively when Chinese patients were given by authoritative figures an opportunity to make their own decision, they might interpret it as an order for admission because they tended to believe that they were expected to give an "appropriate" reply. Therefore being asked to make a choice among Chinese might be perceived as a kind of negative pressure. This cultural difference might explain why items 4 and 7 were loaded on "Negative Pressure Scale".

Current results supported a significant link between C-AES scores and legal status. This corroborated with some previous researches [2-4] while many had reported a partial relationship, with a substantial percentage of voluntarily admitted patients felt being coerced and vice versa [5,13,14,16,30,31]. This may partly be due to the difference in admission procedure or legislation in different settings [16]. One possible explanation for the current result is that the Chinese culture featured a hierarchical relational structure which abides everyone by their respective social roles [29]. As described above, Chinese physicians are expected to offer parental care, and patients tend to comply with medical advices. This might have accounted for less "coerced voluntaries" among those admitted to the hospital.

There was no correlation between C-AES and psychotic symptom severity measured by BPRS. Similar results have been reported in other studies [4,13,30,31]. It is possible that psychotic symptoms or distorted reality testing may not have any direct bearing on the subjective experience of admission or the perceived justification for hospitalization. The admission experience therefore represented discreet constructs that are independent on psychopathology.

The findings of this study need to be considered in the context of several methodological limitations. Firstly, the C-AES was not validated for cognitively impaired patients, and therefore should not be used in this patient group. Secondly, 16 patients were excluded from the study due to capacity issues, illiteracy or visual impairments and three refused to consent. Some of these patients might not be able to understand the nature and purpose of the procedures during their admission, or even lack the capacity to give an informed consent to treatment. They could possibly belong to a particular group who experienced great coercion but have unfortunately been excluded.

Other issues included limitations on assessing the construct validity of the C-AES. Although past studies have shown good criterion validity [2,5,18], the concept of coercion was inherently difficult to be studied because it is unethical to operationalize or manipulate coercion as an independent variable in clinical settings [7]. Unbiased documentation of coercive interaction is equally difficult. This could be explained by the qualitative results of this study that coercion came in all forms of personal interaction, including gesture and use of language. The lack of a suitable gold standard of perceived coercion also hampered the assessment of concurrent validity. Attempt was made to address these issues by identifying the observable indicators of coercion through the use of focus groups. This served as standardized and objective evidence to coercive behaviors for the validation of the construct of admission experience. In addition to the positive association between C-AES scores and OCM, the closest connection between "factual" and "perceived" coercion was observed in item 8 – "Someone physically tried to make me come into the hospital". Among all items of the C-AES, this item had the highest correlation with patients who had been subjected to coercive measures [Table 3]. Nonetheless, OCM and subjective perception of coercion might not be conceptually interchangeable, and discrepancy has been reported [32]. Lastly, the factor loadings in the C-AES exhibited certain discrepancies with the original scale. Future researchers should be aware of these limitations when comparing current results with other studies.

## Conclusion

Current study showed that the C-AES is a psychometrically sound assessment of three aspects of the experience of admission, namely "negative pressure", "process exclusion" and "perceived coercion". Involuntarily committed patients and those subjected to OCM experienced more negative pressure, process exclusion, and perceived coercion than patients voluntarily admitted or not subjected to OCM.

Future research could focus on identifying and reducing the amenable factors that contribute to the perception of coercion, and exploring interventions that enhance patient's involvement in clinical decisions. These are ethical and practical goals that we should work forward to.

## Competing interests

This work was part of the dissertation for completing the HKCPsych Part III Examination 2008. The authors received no financial grants and have no industrial link, affiliations or other potential conflicts of interests.

## Authors' contributions

CKJF participated in the design of the study, carried out the transcription of focus group and interview scripts, administered all patient interviews, performed qualitative and quantitative analysis and drafted the manuscript. PLPC modulated the focus groups. CWLL supervised the research work and finalized the manuscript. All authors read and approved the final manuscript.

## Pre-publication history

The pre-publication history for this paper can be accessed here:



## References

[B1] Monahan J, Hoge SK, Lidz CW, Roth LH, Bennet NS, Gardnes W, Mulvey E (1995). Coercion and commitment: understanding involuntary mental hospital admission. Int J Law Psychiatry.

[B2] Nicholson RA, Ekenstam C, Norwood S (1996). Coercion and the outcome of psychiatric hospitalization. Int J Law Psychiatry.

[B3] McKenna BG, Simpson AI, Laidlaw TM (1999). Patient perception of coercion on admission to acute psychiatric services. The New Zealand experience. Int J Law Psychiatry.

[B4] Poulsen HD (1999). Perceived coercion among committed, detained, and voluntary patients. Int J Law Psychiatry.

[B5] Hiday VA, Swartz MS, Swanson J, Wagner HR (1997). Patient perceptions of coercion in mental hospital admission. Int J Law Psychiatry.

[B6] Day JC, Bentall RP, Roberts C, Randall F, Rogers A, Cattell D, Healy D, Rae P, Power C (2005). Attitudes toward antipsychotic medication: the impact of clinical variables and relationships with health professionals. Arch Gen Psychiatry.

[B7] Gardner W, Hoge SK, Bennett N, Roth LH, Lidz C, Monahan J, Mulvey EP (1993). Two scales for measuring patients' perceptions for coercion during mental hospital admission. Behav Sci Law.

[B8] Seigel K, Wallsten T, Torsteinsdottir G, Lindström E (1997). Perceptions of coercion: a pilot study using the Swedish version of the Admission Experience Scale. Nord J Psychiatry.

[B9] McKenna BG, Simpson AI, Coverdale JH (2006). Outpatient commitment and coercion in New Zealand: a matched comparison study. Int J Law Psychiatry.

[B10] Swartz MS, Wagner HR, Swanson JW, Hiday VA, Burns BJ (2002). The perceived coerciveness of involuntary outpatient commitment: findings from an experimental study. J Am Acad Psychiatry Law.

[B11] Cascardi M, Poythress NG, Ritterband L (1997). Stability of psychiatric patients' perceptions of their admission experience. J Clin Psychol.

[B12] Høyer G, Kjellin L, Engberg M, Kaltiala-Heino R, Nilstun T, Sigurjónsdóttir M, Syse A (2002). Paternalism and autonomy: a presentation of a Nordic study on the use of coercion in the mental health care system. Int J Law Psychiatry.

[B13] Ivar Iversen K, Høyer G, Sexton H, Grønli OK (2002). Perceived coercion among patients admitted to acute wards in Norway. Nord J Psychiatry.

[B14] Kallert TW, Glöckner M, Onchev G, Raboch J, Karastergiou A, Solomon Z, Magliano L, Dembinskas A, Kiejna A, Nawka P, Torres-González F, Priebe S, Kjellin L (2005). The EUNOMIA project on coercion in psychiatry: study design and preliminary data. World Psychiatry.

[B15] Lidz CW, Mulvey EP, Hoge SK, Kirsch BL, Monahan J, Eisenberg M, Gardner W, Roth LH (1998). Factual sources of psychiatric patients' perceptions of coercion in the hospital admission process. Am J Psychiatry.

[B16] Lidz CW, Hoge SK, Gardner W, Bennett NS, Monahan J, Mulvey EP, Roth LH (1995). Perceived coercion in mental hospital admission. Pressures and process. Arch Gen Psychiatry.

[B17] Lidz CW, Mulvey EP, Hoge SK, Kirsch BL, Monahan J, Bennet NS, Eisenberg M, Gardner W, Roth LH (2000). Sources of coercive behaviours in psychiatric admissions. Acta Psychiatr Scand.

[B18] Hoge SK, Lidz CW, Mulvey EP, Roth LH, Bennett NS, Siminoff LA, Arnold RM, Monahan J (1993). Patient, family and staff perceptions of coercion in mental hospital admission: an exploratory study. Behav Sci Law.

[B19] Wertheimer A (1993). A philosophical examination of coercion for mental health issues. Behav Sci Law.

[B20] Bindman J, Reid Y, Szmukler G, Tiller J, Thornicroft G, Leese M (2005). Perceived coercion at admission to psychiatric hospital and engagement and follow-up--a cohort study. Soc Psychiatry Psychiatr Epidemiol.

[B21] Census and Statistics Department, Government of the Hong Kong Special Administrative Region (2006). Population By-census 2006 Hong Kong.

[B22] Hinkin TR (1998). A brief tutorial on the development of measures for use in survey questionnaires. Organizational research methods.

[B23] Hunt SD, Sparkman RD, Wilcox JB (1982). The pretest in survey research: Issues and preliminary findings. J Mark Res.

[B24] World Health Organisation (1992). ICD-10 classification of mental and behavioural disorders Geneva.

[B25] Ventura MA, Green MF, Shaner A, Liberman RP (1993). Training and quality assurance with the brief psychiatric rating scale: "The drift buster". Int J Methods Psychiatr Res.

[B26] Keller J, Gomez RG, Kenna HA, Poesner J, DeBattista C, Flores B, Schatzberg AF (2006). Detecting psychotic major depression using psychiatric rating scales. J Psychiatr Res.

[B27] Costello AB, Osborne JW (2005). Best practices in exploratory factor analysis: four recommendations for getting the most from your analysis. Practical Assessment, Research & Evaluation.

[B28] Hsiao FH, Klimidis S, Minas H, Tan ES (2006). Cultural attribution of mental health suffering in Chinese societies: the views of Chinese patients with mental illness and their caregivers. J Clin Nursing.

[B29] Hui EC (2005). The centrality of patient-physician relationship to medical professionalism: an ethical evaluation of some contemporary models. Hong Kong Med J.

[B30] Cascardi M, Poythress NG (1997). Correlates of perceived coercion during psychiatric hospital admission. Int J Law Psychiatry.

[B31] Kaltiala-Heino R, Laippala P, Salokangas RK (1997). Impact of coercion on treatment outcome. Int J Law Psychiatry.

[B32] Kjellin L, Westrin CG (1998). Involuntary admissions and coercive measures in psychiatric care. Registered and reported. Int J Law Psychiatry.

